# Anisotropic Hydrogel Microelectrodes for Intraspinal Neural Recordings in vivo

**DOI:** 10.21203/rs.3.rs-4693073/v1

**Published:** 2024-08-14

**Authors:** Siyuan Rao, Sizhe Huang, Ruobai Xiao, Shaoting Lin, Eunji Hong, Geunho Jang, Shovit Gupta, Fake Lu, Bo Chen, Xinyue Liu, Atharva Sahasrabudhe, Zicong Zhang, Zhigang He, Alfred Crosby, Kaushal Sumaria, Tingyi Liu, Qianbin Wang

## Abstract

Creating durable, motion-compliant neural interfaces is crucial for accessing dynamic tissues under in vivo conditions and linking neural activity with behaviors. Utilizing the self-alignment of nano-fillers in a polymeric matrix under repetitive tension, here, we introduce conductive carbon nanotubes with high aspect ratios into semi-crystalline polyvinyl alcohol hydrogels and create electrically anisotropic percolation pathways through cyclic stretching. The resulting anisotropic hydrogel fibers (diameter of 187 ± 13 µm) exhibit fatigue resistance (20,000 cycles at 20% strain) with a stretchability of 64.5 ± 7.9%, and low electrochemical impedance (900 ± 149 kΩ @ 1kHz). We observe the re-constructed nanofillers’ axial alignment and a corresponding anisotropic impedance decrease along the direction of cyclic stretching. We fabricate fiber-shaped hydrogels into bioelectronic devices and implant them into wild-type and transgenic Thy1-ChR2-EYFP mice to record electromyographic signals from muscles in anesthetized and freely moving conditions. These hydrogel fibers effectively enable the simultaneous recording of electrical signals from ventral spinal cord neurons and the tibialis anterior muscles during optogenetic stimulation. Importantly, the devices maintain functionality with repeatable recording results over eight months after implantation, demonstrating their durability and potential for long-term monitoring in neurophysiological studies.

### Main

Soft-material solutions have advanced motion-adaptive interfaces to enable neural modulation and recording in vivo, particularly within tissues experiencing complex mechanical dynamics, such as the spinal cord[1-4] and peripheral nerves[5, 6]. These nerve tissues often suffer from damage due to mechanical mismatch between traditional rigid implants and the delicate nervous system, hindering access to deep structures under freely moving behavioral contexts. Therefore, a motion-adaptive soft material neural interface is required to minimize tissue damage during animal natural movement and achieve optimal recording results from physiological signals.

Engineering approaches have been adopted to enhance the electrical functionality of these interfaces. Fractal design concepts provide the capacity to extend conventional silicon-based technologies with soft materials and curvilinear structures[7]. Conductive coatings[8], such as conductive polymers[6, 9, 10] and micro-cracked metallic films[11, 12] applied to soft and elastic substrates, have shown substantial success in electrical stimulation and recording in spinal cord tissue and the peripheral nervous system. The soft and elastic polymer matrix ensures flexibility and stretchability, while the conductive pathways, formed either through the conjugation of polymer backbone or metallic nano- and micro-structures, maintain conductivity under strain. However, most epidural electronics are designed to interface with the surface of the spinal cord, featuring large areas of flexible surface electrodes but lacking access to single-unit neural recordings, especially in the deep ventral structures. Due to the complex anatomical structures and mechanical conditions in the spinal cord, especially in awake and moving animals, the surface coating designs are also limited by the risk of delamination and fatigue under large strains over long-term implantation.

Instead of designing surface epidural electrodes, we hypothesize that penetrating microelectrodes can access deep tissues with greater spatial precision for intraspinal recordings. To achieve stable electrical properties under stretching, embedding conductive fillers directly into the polymeric matrix can create robust, electrically percolative pathways[13, 14]. Particularly, nanocomposite materials containing high-aspect-ratio conductive fillers, allow for the reconstruction of percolation networks through the organization of nanofillers[15]. Such methods address the risk of technical failures of delamination and the diminished overlap of percolation pathways under repeated stretching. Hydrogels show considerable promise as an elastic soft substrate due to their tissue-like mechanical properties (8 kPa–5 MPa)[16] and biocompatibility. Semi-crystalline polymers-based hydrogels, such as cross-linked polyvinyl alcohol (PVA) hydrogel, contain both amorphous polymer chains and polymeric nanocrystalline domains and offer a low elastic modulus and high fatigue resistance under complex mechanical conditions[5, 17]. The integration of conductive nano-fillers within the hydrogel matrix intertwines with polymer chains to create three-dimensional percolative pathways within the elastic substrates.

Both computational and experimental studies have demonstrated that the conductive pathways of nanofillers can be regulated and reconstructed through stretch-induced kinematic movements and self-organization[18, 19]. Additionally, mechanical stretching can induce nanocrystalline re-alignment in hydrogels[17]. In a nanocomposite design, polymeric nanocrystalline support enhances durability and stretchability, while the anisotropic conductive pathways of nanofillers ensure the conductivity necessary for collecting in vivo electrical signals under complex tissue movements. Due to the chemically inert property and high-aspect-ratio structures, carbon-based conductive fillers are particularly suitable for in vivo physiological metabolic environments and the formation of conductive pathways with low concentrations.

In this study, inspired by stretch-induced polymeric nanocrystalline growth and nanofillers reconstruction, we introduced carbon nanotubes (CNTs) into fatigue-resistant PVA hydrogels to create a set of conductive CNTs-PVA hydrogel fibers for electrical recording in vivo. We observed a decrease in hydrogel fibers' impedances after applying cyclic mechanical stretching (5,000 cycles at different strains: 5%, 10%, and 20%) and anisotropic conductivity improvement aligned with the stretching direction after cyclic stretching. Electron microscopy, stimulated Raman scattering microscopy and X-ray examination revealed a Tension Reinforcement for AnIsotropic Nano-orientation (TRAIN) mechanisms underlying the directional conductivity improvement (dispersion degree of CNTs in PVA matrix: 9.60 ± 2.22º). Representative CNTs-PVA hydrogel fibers maintained 64.5 ± 7.9% stretchability and 900 ± 149 kΩ (@ 1kHz) with a diameter of 187 ± 13 μm. For in vivo testing, we fabricated our CNTs-PVA hydrogel fibers into electronic devices and implanted them into mouse hindlimb muscles and spinal cords. In muscle-implanted transgenic *Thy1-ChR2-EYFP* mice, we triggered light-evoked muscle responses and captured electromyographic (EMG) signals through transdermal stimulation with blue light (473 nm) under anesthetized conditions. Moreover, when implanted in the tibialis anterior (TA) and gastrocnemius soleus (GS) muscles, the hydrogel microelectrodes facilitated simultaneous EMG recordings from both muscles during voluntary wheel running tests. With the miniaturized device involving hydrogel microelectrodes being implanted in the spinal cord and hindlimb muscles, we successfully recorded the activities of ventral spinal cord neurons and TA muscle in response to light stimulation in anesthetized ChR2 mice. Remarkably, these intraspinal recording devices demonstrated strong resilience in free-moving conditions, maintaining the capability to record comparable spinal cord neuronal activities in eight months post-implantation. This long-term stability underscores the potential of hydrogel bioelectronics for extended neurophysiological monitoring in naturally behaving animals.

**Stretch-induced conductivity improvement in nanocomposite hydrogel fibers**

Conductive hydrogels have been applied to biosensors[20, 21], wearable electronics[22, 23], and neural interface technologies[24] due to mechanical compliance to complex in vivo conditions. Aiming to examine neuromuscular pathways in the spinal cord and peripheral nerves, we designed semi-crystalline hydrogels that embedded conductive nanofillers to adapt to the dynamic environment in vivo during movements (**Fig. 1a**). Built upon the phenomenon of stretch-induced nanocrystalline domain alignment in hydrogels[5, 17], we utilized the cross-linked PVA hydrogels as the matrix and introduced CNTs with the aspect ratio of 2000–10,000:1 as the conductive nanofillers to create conductive hydrogel microelectrodes for in vivo electrical recordings. Our hypothesis centered on the polymeric nanocrystalline of the hydrogels supporting a durable elastic substrate while the reconstruction of conductive percolative networks through nano-fillers re-alignment provides robust conductivity under stretching (**Fig. 1a-b,**). We incorporated CNTs during hydrogel chemical cross-linking and then subjected CNTs-PVA hydrogels to cyclic stretching (20% strain for 10,000 cycles**, Supplementary Fig. 1**). Scanning electron microscopy results indicated a reconfiguration of CNTs bundle structures, which aligned along the direction of applied strain from (**Fig. 1c**).

To examine whether these microstructural changes correlated to their functional performance, we first characterized the electrochemical impedances of the conductive nanofillers-embedded hydrogel fibers. We prepared the CNTs-PVA hydrogel fibers with a series of CNTs concentrations in the range of 0.04 wt% to 0.24 wt%, incorporated into a 10 wt% PVA hydrogel matrix and examined their electrochemical impedance spectroscopy results (EIS, **Fig. 1d** and **Supplementary**

Fig. 2**). We found that, at a 0.04 wt% CNT inclusion, the impedance measured at approximately 7.09 ± 0.69 kΩ mm at 1 kHz, which is a commonly used frequency for single-neuron activity recording. These results suggest that this nanofiller concentration surpasses the percolation threshold[25-27]. To compare the electrical performance of the CNTs-PVA hydrogel fibers across varying CNT loadings, we normalized the impedance values relative to the cross-sectional area and the effective length of the uninsulated fibers under examination. This analysis revealed a decrease in the specific impedance with an increase in CNTs content, with values declining from 7.09 ± 0.69 kΩ mm to 3.64 ± 0.26 kΩ mm, thereby indicating the role of CNTs in contributing to the conductivity of the hydrogel fibers (**Fig. 1d**).

Then we examined the effect of stretching on CNTs-PVA hydrogels impedance change. We included two types of stretching condition in the evaluation: Employing stretching during the hydrogel drying and annealing processes, which was denoted as "pre-stretch" and cyclic stretching under water when the hydrogel fibers underwent drying, annealing and re-hydration. To compare the stretching effect, we employed a group of CNTs-PVA hydrogel fibers (0.04 wt%, 0.08 wt%, 0.16 wt% and 0.24 wt% CNTs in 10 wt% PVA) with a 200% pre-stretch, a group with the combination of 200% pre-stretch with subsequent cyclic training (denoted as TRAIN hydrogel, 10% strain for 5,000 cycles under water), and a group of unstretched samples as the control (**Fig. 1e**). As a representative example, for fibers with a CNTs loading of 0.16 wt% and a diameter of 187 ± 13 µm, cyclic stretching not only reduced impedance from 5.06 ± 1.02 kΩ mm (unstretched fibers) to 3.00 ± 0.37 kΩ•mm but also yielded a lower impedance than fibers that underwent only a single 200% pre-stretch with the impedance of 3.81 ± 0.41 kΩ mm (**Fig. 1e**). To further explore the impact of mechanical training, we adhered to Hooke's law[28] and defined the first 20% of strain as the elastic zone for establishing training strains in our CNTs-PVA hydrogels and observed a general enhancement in conductivity across all applied strains (**Fig. 1f**). To systematically assess the influence of mechanical stretching with different CNTs loadings, we measured the impedance ratio of the hydrogel fibers before and after 5,000 cycles of stretching and found over 1.03 ratio across all the groups, indicating a consistent effect of the mechanical stretching on the electrochemical impedance improvement (**Fig. 1g**).

In alignment with the reported phenomenon of shakedown after prolonged cycling in tough hydrogels[29], we observed that extending the stretching processes of the CNTs-PVA hydrogel fibers from 20,000 cycles with 20% strains resulted in a steady status after 5,000 cycles (**Fig. 1h**). We measured the electrochemical impedance of each CNTs-PVA fiber and compared these measurements to the original fibers without any stretching. The impedance continued to decrease during the cycling process until it stabilized at approximately 5,000 cycles, maintaining 35.64 ± 18.62 % of the original impedance. We also discovered that the water content faction of TRAIN CNTs-PVA hydrogel fibers reached a steady level at approximately 5,000 cycles (78.6 ± 3.5%, **Supplementary Fig. 3**). Given that hydrogel microelectrodes will undergo repetitive stretching conditions in vivo, maintaining the impedance and water content fraction of CNTs-PVA hydrogel fibers within this steady region is crucial for ensuring the stable performance of hydrogel electrodes in detecting electrophysiological signals.

**Tension Reinforcement for AnIsotropic Nano-orientation (TRAIN) strategy**

To investigate the underlying mechanisms in the observed conductivity improvement after stretching, we measured the morphology change of conductive fillers in hydrogel matrix after stretching treatment. We applied a customized stimulated Raman scattering (SRS) microscopy to the CNTs-PVA hydrogels to visualize the alignment of CNTs within the PVA hydrogel matrix with chemical bond information (**Fig. 2a-b**). SRS employs coherent amplification via a Pump beam and a Stokes beam, resulting in a substantial enhancement of the Raman signal strength compared to the relatively weak spontaneous Raman scattering process[30, 31]. We first detected the featured G-band of CNTs at 1596 $cm^{-1}$ as the indicative C-C bond[32, 33] (**Figs. 2c**). From the SRS microscopy images, we observed that following 10,000 cycles of cyclic stretching at a 20% strain, CNTs within the PVA hydrogels were preferentially aligned along the axis of stretching (**Fig. 2b**, 0°, denoted as TRAIN hydrogels). This realignment contrasted with the random orientation seen in the untrained CNTs-PVA hydrogels (**Figs. 2b** and **d**). To quantitatively assess the realignment of CNTs, we adopted an evaluative method to measure dispersion degree metric[21], and the results indicated a significant reduction in the dispersion degrees in TRAIN hydrogels (9.60 ± 2.22°) as compared to their untrained counterparts (29.32 ± 6.98°) (**Fig. 2e**). These results supported the hypothesis that the cyclic stretching led to the re-alignment of nano-fillers in elastic hydrogel matrix.

Based on morphological observations of the nanofillers, we then explored whether such structural re-alignment leads to axis-dependent impedance changes. We prepared CNTs-PVA films (2 cm × 2 cm × 180 μm, CNTs concentrations of 0.16 wt%) and applied cyclic stretching (10% strain for 10,000 cycles) to assess impedance change along different axes. We defined impedance along three orthogonal vectors: the *x*-axis, aligned parallel to the stretch, and the *y* and *z*-axes, perpendicular to the stretching direction (**Fig. 2f**). To measure the impedance along these respective directions, we designed an electrochemical cell connected to a potentiostat. For the *x* and *y* axes, impedance was measured using two platinum electrodes positioned on the film's surface (**Fig. 2g**, left). Conversely, for the *z*-axis, impedance measurement involved enveloping the film between two platinum films, covering the full area on opposing surfaces (**Fig. 2g**, right).

The impedance of hydrogel films was then normalized based on the distance between electrodes and the contact area of the platinum films. Consistent with our observations in CNTs-PVA hydrogel fibers (**Fig. 1e** and **f**), we observed a 17.43 ± 11.12% impedance decrease, from 12.02 ± 0.96 kΩ mm to 9.88 ± 1.19 kΩ mm along the stretching direction ($x$-axis), with no significant change on the $y$-axis and a 17.49 ± 6.04% increase along the $z$-axis **(Fig. 2h and Supplementary Fig. 4**). Along with the change in impedance, we also found the dimensions in $x$, $y$ and $z$ directions changed post-training, yet the volume of TRAIN CNTs-PVA hydrogel films remained constant (**Supplementary Fig. 5**). Consequently, we linked the evidence of the anisotropic impedance enhancement along the stretching direction and the re-alignment of the conductive nano-fillers CNTs within the PVA matrix, resulting from the re-constructed percolative network.

Similar as previously reported phenomena in semi-crystalline hydrogels[17, 34], we also observed the re-orientation of polymeric nanocrystalline domains in TRAIN hydrogel fibers using Wide Angle X-ray Scattering (WAXS) (**Fig. 2i**). These results indicated a global re-alignment of nanostructures in CNTs-PVA hydrogels under stretching.

**TRAIN hydrogels mechanical properties**

While the conductive nanofiller CNTs contributed to the electrical characteristics of CNTs-PVA hydrogel fibers (**Figs. 1d-g**), we further investigated how the stretching treatment affects the mechanical properties, especially under the consideration for in vivo tissue movements. The single tensile test results revealed that 0.16 wt% CNTs-PVA hydrogel fibers without training exhibited a relatively low elastic modulus of 6.45 ± 1.63 MPa (**Fig. 2j, Supplementary Figs. 6** and **7**), along with a maximum elongation of 124.09 ± 50.46% (**Fig. 2k, Supplementary Fig. 6**). However, the elastic modulus of the fibers significantly increased to 32.74 ± 5.74 MPa post a 200% pre-stretch treatment during drying and annealing, due to the internal-stress induced rigid nanofillers' alignment, similar to previous research[35, 36]. However, we found further cyclic stretching underwater led to a reduction in the elastic modulus to 15.97 ± 5.82 MPa (**Fig. 2j**), potentially attributed to the shakedown phenomenon in hydrogels[29]. Fibers subjected to the TRAIN strategy (20% strain for 10,000 cycles) showed an elastic modulus comparable to that of the untrained fibers, indicating that the TRAIN method did not compromise the inherent softness of the PVA hydrogel, an advantageous feature for motion-adaptive in vivo applications. To delve deeper into the effects of CNTs on the PVA hydrogel nanostructures, we employed Differential Scanning Calorimetry (DSC) to characterize the crystallinity within the TRAIN hydrogel fibers. A shift in the characteristic endothermic peak of PVA nanocrystals was observed, and upon quantifying the enthalpy changes for different CNTs concentrations (0.16 wt% and 0.24 wt%), we deduced that CNTs could modulate nanocrystalline growth within the PVA matrix[34], reducing crystallinity from 26.5% to 11.5% (**Fig. 2l**). This observation aligns with the report of introducing nano-fillers inhibits the nano-crystallization in hydrogels[37].

#### Electromyographic (EMG) recording with TRAIN microelectrodes in moving mice

To facilitate in vivo animal studies, we fabricated thin CNTs-PVA hydrogel fibers (187 ± 13 μm in diameter, **Fig. 3a**) into electrical devices with a layer of styrene-ethylene-butylene-styrene (SEBS) thermoplastic elastomer as an insulating coat via the dip-coating method (thickness: 1.8 ± 0.4 μm). Next, we surgically implanted hydrogel microelectrodes into the TA and GS muscles of the hindlimbs in *Thy1-ChR2-EYFP* mice (**Figs. 3b** and **c**). We chose these transgenic mice because they intrinsically express the photo-excitable Channelrhodopsin 2 (ChR2) within their nervous systems, which facilitates neuronal manipulation and recording from live animals. We first confirmed the expression of ChR2 in mouse sciatic nerves through confocal microscopy (**Fig. 3d**).

With anesthetized *Thy1-ChR2-EYFP* mice, we applied blue light stimulation (473 nm) transdermally to the hindlimb muscles, inducing neural excitation and subsequent muscle contraction (**Fig. 3e**). Before implantation, the TRAIN hydrogel electrodes were dehydrated to enhance stiffness, facilitating easier insertion. Once implanted, the electrodes rehydrate from tissue fluids, softening to integrate more seamlessly with muscle tissues. We found that the TRAIN hydrogel electrodes consistently captured EMG signals in both the TA and GS muscles correlated well with pulsed transdermal blue light stimulation (473 nm, 0.5 Hz, pulse width 50 ms). These EMG recordings demonstrated uniform amplitudes and waveform and exhibited high signal-to-noise ratios (SNR: 9.25 in TA), attesting to the electrodes' efficacy (**Figs. 3f** and **g**).

After confirming TRAIN microelectrodes' functionality in vivo, we further tested their performance in conditions involving naturalistic animal movement. As a proof-of-concept application, we collected mouse EMG signals with TRAIN microelectrodes during voluntary wheel running tests (VWRTs)[5, 38], a standard assay for assessing locomotor activity by labeling skeletal landmarks with a network of nodes to track the running behaviors through a markerless motion tracking algorithm, DeepLabCut[5, 39]. We first evaluated whether the implantation of TRAIN microelectrodes in mouse TA and GS muscles impaired mouse natural movement by performing gait analysis on implanted hindlimbs. Three days after the mouse recovery from the implantation surgeries, we compared the gait of the implanted mice to the sham controls in their gait kinematics during swing and stance phases, which showed no significant differences (**Fig. 3h** to **j**), indicating surgical implants do not adversely affect mouse basic gait parameters.

With the implanted microelectrodes remaining in the muscles over 1-week post-implantation, we conducted EMG recordings from TA and GS muscles simultaneously by implanting TRAIN hydrogel microelectrodes in both sites while mice moved freely during VWRT and correlated the collected EMG signals to locomotion behaviors (**Fig. 3k, left**). By synchronizing the EMG recordings with locomotion behaviors, we observed a clear alternative EMG activity of TA and GS muscles (**Fig. 3k, right**), in line with previous studies that used conventional electrode EMG recordings[40-42].

**Intraspinal electrophysiology recording with TRAIN microelectrodes**

Leveraging the TRAIN hydrogel microelectrodes can detect EMG signals in freely moving animals, we further explored whether it could detect neuronal electrical activity in live spinal cords. We integrated three TRAIN microelectrodes into one miniaturized device (**Fig. 4a**) to detect electrical recordings simultaneously from mouse spinal cords and muscles. We implanted the miniaturized device into the mouse back region, with one electrode into the Lumbar (L) 3 spinal cord ventral horn region, where the TA muscle motor neurons are located, and the other one into hindlimb TA muscle in *Thy1-ChR2-EYFP* mice (**Fig. 4b** and **c**). Following a three-day recovery, the *Thy1-ChR2-EYFP* mice were subjected to transdermal optical stimulation (473 nm, 0.5 Hz, pulse width 50 ms) under anesthesia, with simultaneous electrical recordings from both ventral horn part of the L3 spinal cord and the TA muscle **(Fig. 4d**). To verify that the recorded electrical signals from the spinal cord and TA muscles came from the transdermal optogenetic stimulation, we altered the intensity of the blue light pulses and compared the corresponding electrical signal changes. By isolating and overlaying each detected electric peak (n=10 individual peaks), we noted distinct changes in the amplitudes and waveforms of the extracellular signals from the spinal cord and the EMG signals **(Fig. 4d**), which underscored the responsiveness of hydrogel microelectrodes.

Given these promising results, we assessed their capability to detect spontaneous neural activities from the spinal cord and stay functional over long-term observations. We first recorded for the endogenous neural spikes from anesthetized mice 1-month post-implantation with a bundle of three TRAIN hydrogel microelectrodes implanted in ventral horn at L3 (**Fig. 4e**). Single neuron activities were delineated using principal component analysis (PCA) (**Fig. 4f**). Then we examined the electrodes' performance in awake mice by recording from the same group of mice using the same device. We observed the sorted neural spikes maintained the same waveforms but with a higher firing rate (FR), from a representative FR of 32.60 Hz under anesthesia to an FR of 51.33 Hz during awake (**Figs. 4g** and **4h**). We also found that the TRAIN hydrogel electrodes maintained long-term stability and durability in vivo with the electrophysiological recording from mice 8 months post-implantation. We found that the implanted hydrogel microelectrodes were still able to collect spontaneous neural activities in the mouse spinal cord with distinct single-unit waveform and amplitude (**Fig. 4i-l**). When the implanted mice were set to freely moving (**Fig. 4m**), we observed a combination of burst patterns with large electrical peak clusters along with movement and tonic spikes (**Fig. 4n**), indicating the adaptivity and flexibility of TRAIN hydrogel electrodes for electrophysiological recordings during in vivo dynamic environments.

Beyond the traditional spinal cord surface epidural recording devices[43-47], fiber-shaped or other penetrating microelectrodes demonstrate a capability to probe deep structures of the spinal cord with single-unit precision[48, 49], however, the complex motion and the fragile spinal cord tissues require further soft neural-materials interface designs to minimize tissue damage from motion and the proper motion artifact management[50]. Fatigue-resistant soft hydrogels offer motion-adaptive advantages by improving material-tissue mechanical matching and stretchability. In addition to the compliant interfaces, the reinforced electrical percolative networks support stable interface impedance and consistent conductive pathways to collect electrophysiological signals during dynamic conditions Moreover, such soft bioelectronics also allow minimal disruption to the natural behaviors of the experimental subjects. Using an integrated bioelectronic device to simultaneously record different sites of muscles and nervous systems in the context of behavioral tests, this technology offers direct links between nerve and muscle circuit activities.

### Conclusion

In this study, we engineered a set of hydrogel microelectrodes with a bottom-up approach to reinforcing the percolative network of nanofillers and therefore provided adaptive tissue-material interfaces. Such hydrogel microelectrodes enable electrophysiological recording from mouse muscles and lumbar spinal cord ventral horn region under complex mechanical dynamics in vivo. We discovered that the CNTs-PVA hydrogel microelectrodes exhibited anisotropic electrochemical impedance and investigated the mechanism of stretch-induced reconstruction of conductive nanofillers in a hydrogel matrix. With the retained softness and stretchability, as well as the directional conductivity, when we applied these hydrogel microelectrodes in vivo, they robustly collected the electrical signals from multiple muscles simultaneously during mouse locomotion. Using an integrated device with hydrogel microelectrodes implanted in the mouse ventral horn of the spinal cord and muscle separately, we captured the light-evoked electrical signaling in the sciatic-spinal-motor reflex arc with transgenic mice. Due to the strong motion adaptation to the spinal cord tissues, we successfully recorded the electrophysiological signals from mouse ventral horn areas from various statuses, such as under anesthesia, awake, and naturally behaving. This soft nanomaterials-supported tissue-integrated bioelectronics offers a solution for collective recording from multiple sites, especially in those with severe mechanical dynamics, and a holistic understanding of neural circuits in the context of behaviors.

### Methods

#### Fabrications of conductive hydrogel fibers

To prepare a 10 wt% polyvinyl alcohol (PVA) solution, 10 g of PVA (146,000 to 186,000 Da, 99+% hydrolyzed, Sigma Aldrich 363065) was combined with 90 g of Milli-Q water (14 MΩ·cm at 25 °C). The mixture was heated to 100 °C under continuous stirring for 5 hours, resulting in a clear and viscous solution. For the carbon nanotubes (CNTs) preparation, a stock solution of CNTs (OCSiAl, TUBALL BATT H2O 0.8% beta) was diluted with Milli-Q water in a weight ratio of 1:0.6. To prevent the aggregation of CNTs, a solution of Sodium Dodecylbenzene Sulfonate (SDBS, 95%+, Fisher Scientific D0990500G) was prepared at a concentration of 5 wt%. To fabricate chemically cross-linked CNTs-PVA hydrogel fibers, 100 µL of Glutaraldehyde (GA, 25% in water, Sigma Aldrich G6257) was added into 10 g of 10 wt.% PVA solution followed by mixing and degassing in a vacuum spinner (Musashi ARV-310). 150 µL of hydrochloric acid (HCl, 37%, Sigma-Aldrich 258148) was then added into another 10 g of 10 wt.% PVA solution with mixing and degassing followed. Next, the diluted CNTs, PVA-GA, and PVA-HCl solutions were combined on a ratio of (0.6~1.4):1:1 with the same mixing and degassing procedure. The homogeneous solution was then infused into silicone molds (800 µm I.D., 51845K51 and 500 µm I.D., 51845K66, MacMaster-Carr) and allowed to cross-link at room temperature (RT) for 2 hours. Dichloromethane (DCM, 99.8%, Sigma Aldrich 270997) was utilized to induce swelling in silicone molds, aiding the elution of CNTs-PVA hydrogel fibers. Post-elution, these fibers were rinsed with large amounts of Milli-Q water for 2 days to remove any residual unreacted chemicals.

Following the washing step, the CNTs-PVA hydrogel fibers were immersed in HCl solution (12 mM) for 2 hours (cite COMPACT), then pre-stretched to 200% strain and dried in the air for 12 hours followed by annealing at 100 °C for 20 minutes. Finally, the fibers were reswelled in Milli-Q water for later use.

**Fabrications of conductive hydrogel films**

The CNTs stock solution was diluted with Milli-Q water (1:0.6 by weight) and then homogeneously dispersed with a 5 wt% SDBS solution (1:10 by weight). Subsequently, 100 μL of GA was incorporated into 10 g of a 10 wt% PVA solution. Additionally, 150 μL of HCl was added to another 10 g of 10 wt% PVA solution. Both mixtures were subjected to thorough mixing and degassing using a vacuum spinner. The diluted CNTs, PVA-GA, and PVA-HCl, were then combined in a ratio of 1.4:1:1, employing identical mixing and degassing procedures. The resultant homogeneous mixture was poured into a customized mold and allowed to cross-link at room temperature for 2 hours. The resultant CNTs-PVA hydrogel films were demolded and extensively rinsed with Milli-Q water to eliminate any unreacted chemicals. The films underwent a post-treatment with an HCl solution (12 mM) followed by pre-stretching the films to 10% strain and air drying for 12 hours. The films were annealed at 100 °C for 20 minutes. Finally, these films were reswelled in Milli-Q water.

**TRAIN hydrogel materials**

To reorient CNTs in the PVA hydrogel matrix and fabricate TRAIN hydrogel fibers and films, we subjected them to cyclic stretching tests (strain-controlled mode: 5%, 10% and 20% strains, and frequency of 0.5 Hz) for 5, 000-15,000 cycles using a horizontal mechanical tester (Univert, Cell

Scale) with a water bath. A 4.4 N load cell (Futek) was used. The conductive hydrogel materials were stretched with a constant strain amplitude, and the force variation was recorded over time.

**Electrochemical characterization of conductive hydrogel materials**

Impedance of conductive hydrogel fibers and thin films was systematically assessed using an electrochemical working station (Princeton Applied Research PARSTAT 2273). A 3-electrode electrochemical sink was used to characterize conductive TRAIN hydrogel fibers with a sinusoidal driving voltage spanning a frequency range from 10 Hz to 1 MHz and an amplitude of 10 mV. The conductive hydrogel films, after undergoing repeated training, were analyzed using a specialized device in three different orientations: along the stretching direction (*x*-axis), across the stretching direction on the surface of the film (*y*-axis), and through the thickness of the film, perpendicular to the stretching direction (*z*-axis). For measurements along the *x* and *y* axes, impedance was determined using two platinum wires (diameter: 1.54 mm, Length: 19.47 mm, and distance 15.44 mm). The length ($l$) used in calculations is the 15.44 mm distance between the wires, and the area ($A$) is the contact area where each wire touches the film. For the Z-axis measurements, impedance was recorded using two platinum sheets (length: 19.47 mm and width: 6.41 mm). Here, $l$ is the thickness of the film, and $A$ is the area where each sheet contacts the film. For hydrogel fibers, $l$ is defined as the length of fiber immersed in a chemical solution, and $A$ is the cross-sectional area of the immersed part of the fiber. The lengths of the hydrogel fibers and films were measured using a caliper, while their thicknesses were accurately gauged using a micrometer. The measured impedance was normalized using the specific formula: $R = \frac{l}{A}\rho$, where $R$ is the measured impedance, $l$ is the length of the materials that were characterized, $A$ is the area of the materials that were characterized, $\rho$ is the specific impedance of the conductive hydrogel materials.

**Dimension measurements of hydrogel fibers**

Images of hydrogel fibers were captured using a microscope (AmScope) under a bright field while submerged in water. Each fiber was examined in three distinct areas, including both ends and the middle (three individual measurements in each section). Subsequently, the Image J software was used to measure the diameter of each fiber. In addition, the length (five individual measurements) of the fibers was measured using a caliper.

**SEM characterization of TRAIN hydrogel films**

To investigate the structure and morphology of hydrogel films, samples were first rapidly frozen using liquid nitrogen to preserve their internal structure. For TRAIN conductive hydrogel films, they were fractured along lines parallel to the training direction to expose the relevant structural facets. For pre PVA hydrogel films, they were fractured randomly. After fracturing, the hydrogel films were sputtered with platinum to enhance conductivity and then examined using SEM (Hitachi Su5000) at an acceleration voltage of 4 kV. For representative SEM images, each sample was repeated ten times with similar results.

**TEM imaging of CNTs**

TEM images were acquired using a transmission electron microscope (FEI Tecnai 12, 120 kV). CNTs were diluted in a range from 1:3.3 to 1:10 with Milli-Q water and subsequently deposited onto a copper grid (Sigma-Aldrich FCF200-Cu) for imaging purposes. To ensure the reproducibility of the observations, each sample was imaged 6 times, consistently yielding similar results.

**Raman Spectroscopy**

Raman spectra of TRAIN CNTs-PVA and PVA hydrogel films were acquired using a confocal Raman microscope (Renishaw InVia, 785-nm NIR laser, 20X objective lens). Prior to spectral collection, the films were mounted on glass slides enveloped in a reflective substrate. The acquisition resolution was 0.7 $cm^{-1}$ and the integration time per acquisition was 10 seconds. For the TRAIN CNTs-PVA hydrogel films, to prevent thermal degradation of the CNTs, the laser power was reduced to 0.1%, resulting in an effective power of approximately 0.57 mW at the focal point of the sample. In contrast, the PVA hydrogel samples were analyzed under 10% laser power, corresponding to an effective power of about 10 mW at the sample focus. All spectra were recorded and subsequently analyzed using Renishaw's WiRE 4.1 software, ensuring precise and reproducible data for further interpretation.

**SRS microscopy**

To prepare samples for SRS imaging, TRAIN and untrained CNTs-PVA hydrogel films (0.16% and 0.24% CNTs) were imbedded in O.C.T compounds (-80 $^{o}C$ for 2 hours) and then sliced with a 20 μm thickness using a cryo-slicing microtome (Leica CM1950). The sliced samples were then mounted onto glass slides for later imaging. A customized SRS setup was used to image for specific CNT signals in the TRAIN and untrained CNTs-PVA hydrogel samples. Insight X3, (Spectra-Physics), a dual channel/wavelength, pulsed laser was used to generate the SRS signals. The modulated SRL signal (stimulated Raman loss) was captured with FDS1010 Si-photodiode (Thorlabs Inc.), which was then processed/demodulated by a HF2LI lock-in amplifier (Zurich Instruments). Scan image software was used as an overall image-capturing system to generate the final digital SRS images with 1024X1024 pixel data density. Imaging was performed under a 25X water immersion objective with a 3X zoom, capturing an effective sample area of 233x233 $\mu m^2$. The strokes and pump beams were set to an average power of 100 mW and 30 mW, respectively. The strokes beam is fixed at 1045 nm, and the tunable pump beam was set to 805, 809, 880 and 896nm to obtain 4 sets of SRS images for each sample and location. SRS images were taken at 896 nm for the CNTs selective chemical imaging (based on Raman spectra) and 805 nm for the PVA hydrogel imaging. Images were also taken at 88 0nm and 809 nm for off-peak (background) values, of CNTs and PVA hydrogel respectively. The collected images were processed, background subtracted, and overlaid with pseudo-colors for visualization, using Fiji ImageJ software. To ensure the reproducibility of the observations, each sample was imaged 6 times, consistently yielding similar results.

**Mechanical tests of conductive hydrogel fibers**

Tensile test machine (Univert, Cell Scale) was employed to stretch the hydrogel fibers a rate of 1 mm/s under water. The nominal stress was calculated using the formula $\sigma=F/A$, where $F$ denotes the recorded force and $A$ denotes the cross-sectional area of the fibers in the hydrated state. The strain was calculated using $\epsilon=\Delta L/L$, where $\Delta L$ represents the displacement, and $L$ represents the gauge length. The elastic moduli € were determined by calculating the average slope of the stress-strain relationship in the first 10% of applied strain using linear regression. The maximum elongation (%) of the fibers was reported at the point of fracture in the stress-strain curve.

**X-ray scattering**

X-ray scattering experiments were conducted using the SAXSLAB GANESHA 300XL instrument, equipped with a Dectris Pilatus 300K 2D CMOS photon counting detector (measuring 83.8 x 106.5 $mm^2$). Wide angle X-ray (WAXS) measurements employed a 2mm beamstop, and each sample was exposed for a duration of 300s.

**Measurement of crystallinities**

The degree of crystallinity of hydrogel fibers and materials was assessed using a DSC instrument (2920 TA instrument). The PVA hydrogels were analyzed in the desiccated state. A small quantity of sample (1-15 mg) was loaded into a crucible (TA instrument T81006) and placed in a temperature-controlled DSC cell. A blank crucible served as a reference. The sample was heated from 30 °C to 300 °C in air, with a heating rate of 20 °C/min. The differential heat flow to the sample and reference was recorded by the instrument. To determine the melting fusion enthalpy of endothermic peaks, heat flow (mW) over sample weight (mg) was plotted against time (s). The areas of melting endothermic peaks were integrated using TA analyze software (TA Universal Analysis). The degree of crystallinity α was estimated using the equation: $\alpha=\Delta H_f/\Delta H_m \cdot 100\%$, where $\Delta H_f$ (J/g) was calculated from the integration of melting endothermic peaks and $\Delta H_m$ (150 J/g) was the enthalpy of melting 100% of PVA crystallites (cite).

**Assembling of conductive hydrogel bioelectronic device**

To fabricate TRAIN hydrogel electrodes, a hydrogel conductive fiber and a stainless-steel wire were dip-coated in CNTs (0.8 wt%) solution. The dip-coated hydrogel fiber and stainless-steel wire were inserted into an elastic tubing (100 µm) to create a hydrogel-steel junction. Another thin layer of silver paint (xx) was applied at the junction to enhance conductivity. UV epoxy (Norland optical adhesive, #) was used to seal and reinforce the junction. To insulate the TRAIN hydrogel electrodes, Styrene-ethylene-butylene-styrene (SEBS, 20 wt.%) was diluted in Toluene (Fisher Scientific T290-1) to form a uniform solution. The TRAIN hydrogel electrodes were dipped into the solution and allowed to dry in the air for 1 hour. To assemble the bioelectronic device for EMG recordings, 4 insulated TRAIN electrodes were soldered onto 4 pins from a 6-pin connector (xx, #), respectively. 1 insulated stainless-steel wire (50 μm) was soldered onto the last pin as the ground wire. To assemble the bioelectronic device for spinal cord recordings, 3 insulated TRAIN electrodes were soldered onto 3 pins from a 4-pin connector, respectively. 1 insulated stainless-steel wire (50 μm) was soldered onto the last pin as the ground wire. The 3 working electrodes were twisted into a yarn and dipped coated in SEBS solution. Optical adhesive was applied to the soldering sides on the pin to provide insulation.

**Experimental animals**

All experiments on mice were reviewed and approved by the The Institutional Animal Care and Use Committee at Binghamton University (Protocol number: 897-23) and University of Massachusetts Amherst (Protocol number: 2520). Wild-type (C57BL/6J) and *Thy1-ChR2-EYFP* mice were purchased from the Jackson Laboratory. Mice were given ad libitum access to food and water and were housed at 24 °C ± 1 °C, with 50% relative humidity, and on a 12-h light/12-h dark cycle. All experiments were conducted during the light cycle.

**In vivo implantation in muscle for anesthetic EMG recording**

*Thy1-ChR2-EYFP* mice were anesthetized using isoflurane (1.5% induction) and continuously maintained at 1%. Supplemental heat was provided during the surgery. The fur was removed over the hindlimbs. To collect EMG signals from *Thy1-ChR2-EYFP* mice under anesthesia, an insulated TRAIN hydrogel electrode was inserted into the medial tibialis anterior (TA) and gastrocnemius (GN) muscles of mice hindlimbs. A reference needle electrode was inserted in an electrical unrelated region. A ground needle electrode was subcutaneously inserted into the tail. A 473 nm laser was used for transdermal optical stimulation. EMG data triggered by optogenetic activation were filtered (10 Hz to 1 kHz) collected through a DAM50 system.

**In vivo implantation in muscle for freely moving EMG recording**

C67BL/6J mice were anesthetized with 1.5% isoflurane continuously maintained at 1%, with supplemental heat provided throughout the surgical procedure. Fur was removed from the hindlimbs and head, and the exposed skin was sanitized using iodine followed by 75% ethanol. Incisions were then made at each site.

A 6-pin device was carefully routed subcutaneously from the hindlimb to the skull. The pin connector was securely affixed to the skull using dental cement (Parkell C&B METABOND and Jet Set-4). In wild type mice, two TRAIN hydrogel electrodes were employed—one as a working electrode and the other as a reference electrode. Both electrodes were inserted into the mid-belly of the tibialis anterior (TA) muscles on the left hindlimb using 30-gauge needles. Similarly, two additional TRAIN hydrogel electrodes were inserted into the gastrocnemius (GS) muscle on the left hindlimb of the same wild type mice. A ground wire was positioned subcutaneously in the neck-shoulder area to complete the setup. The skin over the hindlimb muscles was then sutured closed using nylon sutures (Nylon 5-0).

**In vivo implantation in spinal cord**

*Thy1-ChR2-EYFP* mice were anesthetized using 1.5% isoflurane for induction and continuously maintained at 1%, and supplemental heat was provided to maintain body temperature during the surgery. The fur on the dorsum was shaved, and the skin was subsequently disinfected with povidone iodine followed by 75% alcohol. A single midline incision was made over the vertebral segments from T11 to T13, and the paraspinal muscles were dissected away to expose the underlying vertebrae. Using lateral spinal clamps, the exposed section of the spine was stabilized on a stereotaxic frame between T11 and T13. Further dissection exposed the spinal cord between the L3 and L4 vertebrae. An electronic device was then carefully positioned on the dorsal surface of the spinal cord and lowered by 400 μm into the dorsal horn to ensure precise placement. The device was secured using dental cement over the T12 and T13 vertebrae to prevent displacement (Parkell C&B METABOND and Jet Set-4). Closure of the surgical site was achieved by suturing the skin over the dorsum using Nylon 5-0 sutures.

**Synchronized EMG recordings**

*Thy1-ChR2-EYFP* mice were anesthetized using isoflurane (1.5% induction and continuously maintained at 1%). The fur was removed over the hindlimb. One TRAIN hydrogel electrode was inserted in the TA muscle, and one TRAIN hydrogel electrode was inserted into the GS muscle. One needle reference electrode was inserted in the unrelated region, and one needle ground electrode was inserted in the tail. The working electrodes (TA and GN), reference electrode and ground electrode were connected to a DAM8 system. Transdermal optical illumination on the sciatic nerves was carried out using a 473 nm laser The laser was pulsed at a frequency of 0.5 Hz with a pulse width of 50 ms during optical stimulation. Signals were digitized at 4 kHz (DI-1100, DATAQ Instruments) and filtered between 1-1000 Hz. The amplitude and noise level of evoked potentials were assessed utilizing a MATLAB algorithm incorporating a bandpass filter ranging from 0.1 to 300 Hz.

**Running wheel behavioral assay for freely moving EMG recordings**

All the mice were acclimatized to a running wheel for 30 mins, 1 day prior to surgery. 3 days after the implantation of the TRAIN hydrogel electrodes, mice were connected to a DAM50 system through wires and acclimatized to the running wheel for 30 mins. The EMG data was filtered (100-3000 Hz) and collected through a recording system (PowerLab 4/20T, ADInstruments). Concurrently, a camera was used to record videos for the analysis of the gait, using DeepLabCut (DLC), of the mice during locomotion. Critical anatomical landmarks on the mice, encompassing the electronic pin, neck, back, iliac crest, hip, knee, ankle, front toe, and rear toe, were meticulously tracked during the mice's locomotion in the recorded videos. Using a custom-written MATLAB algorithm, we identified a single gait cycle in variable-width runway tasks (VWRTs), defined by the two local minima observed in the plot of the distance between the fore toe and hind toe over time. This gait cycle was further segmented into swing and stance phases. From these data, kinematic stick diagrams representing the hindlimb movement during a single gait cycle were generated, providing detailed insights into the locomotor dynamics of the subjects.

**Synchronized EMG and spinal cord electrophysiological recordings**

*Thy1-ChR2-EYFP* mice were anesthetized using isoflurane (1.5% induction and continuously maintained at 1%) and the implanted 4-pin electronic device at the spinal cord was connected to a DAM8 system through wires. A TRAIN hydrogel electrode was inserted into the TA muscle in the hindlimb, a reference electrode was inserted into the right body of the mice, and a ground electrode was inserted subcutaneously into the tail. A 473 nm laser was used for transdermal optical stimulation over the hindlimb. Spinal cord electrophysiology and EMG data triggered by optogenetic activation were synchronized, filtered (10 Hz to 1 kHz), and collected through the DAM8 system.

**Endogenous recordings in spinal cord under anesthesia**

The spinal cord implanted mice were anesthetized using isoflurane (1.5% induction and continuously maintained at 0.5%). The 4-pin device was connected to a DAM50 system, and the spontaneous neural activities were filtered (100-3000 Hz) and collected through a recording system (PowerLab 4/20T, AD Instruments).

**Open field behavioral assay for freely moving spinal cord recordings**

Prior to recordings, all mice were acclimatized to an open field chamber for 30 minutes to minimize stress and adaptation effects. The same 4-pin device used in the anesthesia studies was connected to a DAM50 system for continuity in data collection. Spontaneous neural activities were filtered across a bandwidth of 100-3000 Hz and collected through the recording system (PowerLab 4/20T, AD Instrument). Concurrently, mouse behaviors were monitored via a camera strategically positioned above the open field chamber, allowing for the correlation of neural activity with observable behaviors.

**Tissue collection**

Once mice were euthanized, their sciatic nerves and spinal cords were removed from the body, washed briefly in PBS (Sigma-Aldrich P3813) and fixed in 4% paraformaldehyde (PFA, Sigma-

Aldrich 8187151000) for more than 48 h at 4 °C in the dark. For confocal microscope imaging, fixed tissues were then placed in 30% sucrose in PBS overnight at 4 °C in the dark. Next, the tissues were embedded in Tissue-Tek O.C.T compound (Sakura Finetek 4583) and cut into 20-μm transverse sections using a cryostat microtome (Leica CM1900).

**Confirmation of transgenic expression**

The tissue sections were permeabilized using PBST (0.3% Triton-X-100 in PBS, Sigma-Aldrich 93443) for 15 minutes at RT. This was followed by blocking with 1% bovine serum albumin (Sigma-Aldrich A9647) in PBS for 30 minutes to prevent nonspecific binding. Subsequently, the tissues were washed three times with PBS and allowed to air dry for 30 minutes. For visualization, the sections were mounted using DAPI-containing Fluoromount-G medium (Southernbiotech, 0100-01) under a cover glass placed on the glass slide. The slides were then left to dry overnight at room temperature. Imaging was performed using a confocal microscope (ZEISS LSM 880). This procedure was repeated ten times for each sample to ensure reproducibility, consistently yielding similar results.

**Statistics and reproducibility**

Statistical analyses were performed in Excel (Microsoft Corporation, Inc., USA), MATLAB (MathWorks, Inc., USA), GraphPad prism 10 (GraphPad by dotmatics), and DeepLabCut (Mackenzie Mathis). The data was tested with normality distribution before statistical analysis. Power analyses for determining sample sizes were not performed; instead, the group sizes were chosen based on previous research. This enabled direct comparison of our results with the earlier work. Statistical significance was determined by the analysis of One-way ANOVA and Paired t-test.

### Conflicts of interest

The authors declare no conflicting interests.

### Acknowledgments

We are appreciative of Y. Liu for his advice on the electrochemical characterization, H. Kim for her guidance on mechanical property characterization, and D. Matuwana for her proofreading of our manuscript. This study received funding from the National Institutes of Health (R00MH120279), the National Science Foundation Faculty Early Career Development Program (CAREER, 2239030), the Brain & Behavior Research Foundation Young Investigator Grant (29878), Faculty Startup Funds from Binghamton University and UMass Amherst, the UMass Amherst Faculty Research Grant (P1FRG0000000295), the UMass OTCV Technology Development Fund, and the Binghamton University S3IP Small Grant award (ADLG267). Furthermore, we acknowledge the essential resources provided by the Binghamton University core facilities, specifically the Electron Microscopy, X-Ray Scattering, and Animal Care Service departments.

### Author contributions

S. R. and Q. W. conceptualized the anisotropic conductive hydrogels. S. H. and R. X. led the fabrication and characterization of hydrogel materials. S. H., S. G. and F. L. carried out Raman spectroscopy and stimulated Raman scattering analyses. T. L., A. S. and K. S. assisted with the hydrogel nanocomposite synthesis, device fabrication, and electrochemical characterization. S. L., X. L., S. H., A. C. and S. R. designed and conducted the cyclic stretching experiments, material characterization, and data analyses. S. H., B. C., Z. Z., Z. H., S.R. and Q. W. designed and conducted the in vivo tests and data analyses. G. J. conducted the gait analysis. E. H. conducted the tissue processing and analysis. S. H., R. X., Q.W. and S. R. prepared the manuscript with the input from all authors.

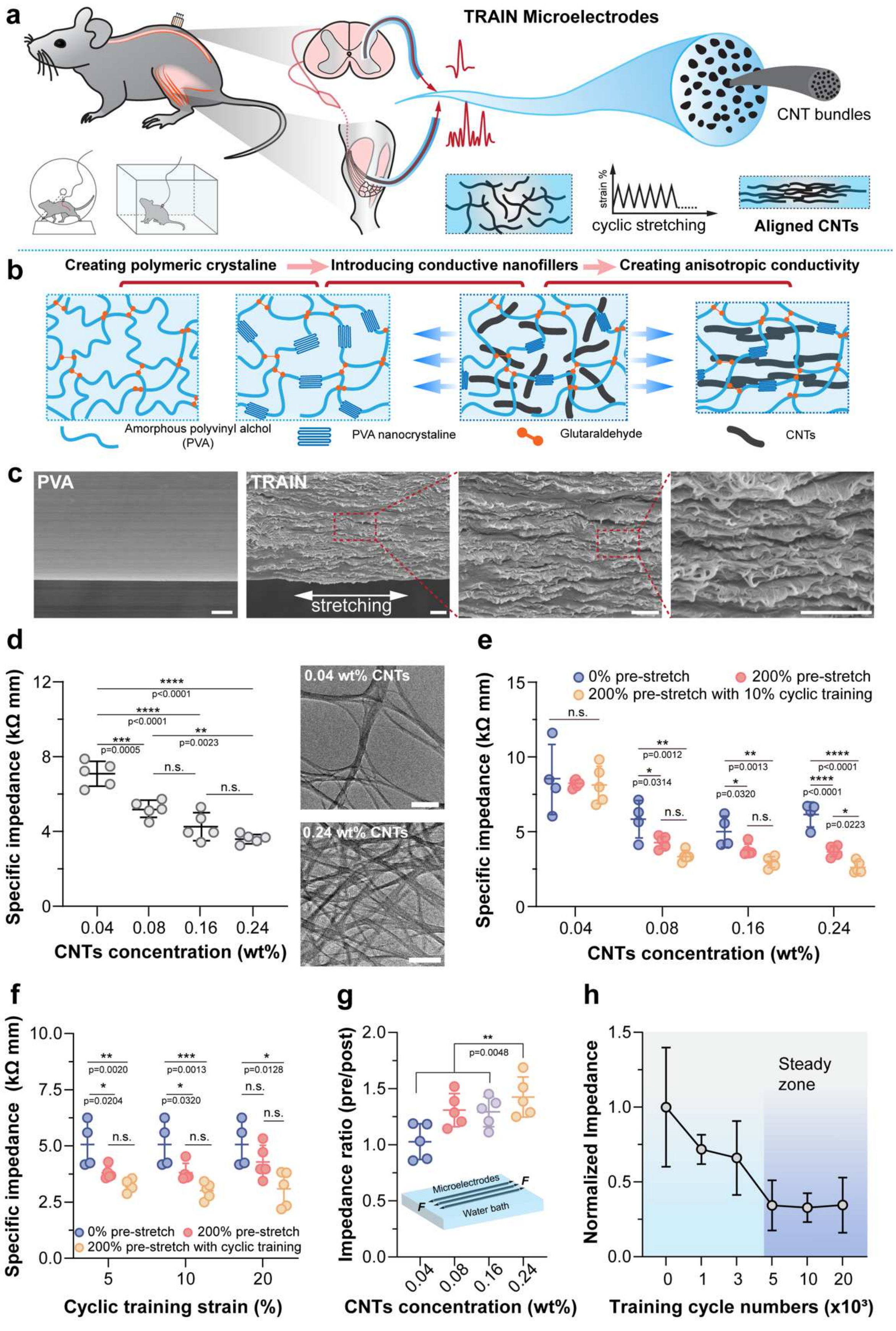

a
TRAIN Microelectrodes
CNT bundles
strain %
cyclic stretching
Aligned CNTs
b
Creating polymeric crystaline
Introducing conductive nanofillers
Creating anisotropic conductivity
Amorphous polyvinyl alchol (PVA)
PVA nanocrystaline
Glutaraldehyde
CNTs
c
PVA
TRAIN
stretching
d
Specific impedance (kΩ mm)
CNTs concentration (wt%)
0.04 wt% CNTs
0.24 wt% CNTs
e
0% pre-stretch
200% pre-stretch
200% pre-stretch with 10% cyclic training
f
Specific impedance (kΩ mm)
Cyclic training strain (%)
0% pre-stretch
200% pre-stretch
200% pre-stretch with cyclic training
g
Impedance ratio (pre/post)
Microelectrodes
Water bath
CNTs concentration (wt%)
h
Normalized Impedance
Steady zone
Training cycle numbers (x10³)

658

**Figure 1. TRAIN strategy for enhancing conductivity in hydrogel fibers. a**, Schematic of the in vivo application of TRAIN microelectrodes, illustrating their implantation into muscle tissue and spinal cord with correlated electrical signals corresponding to muscle and the spinal cord. The TRAIN microelectrode was achieved by the alignment of carbon nanotube (CNTs) bundles in polyvinyl alcohol (PVA) hydrogel through cyclic stretching. **b**, Schematics of microstructures in PVA hydrogels under different phases: chemical cross-linking, nanocrystalline growth, cyclic training after incorporation of CNTs in PVA, and realignment of CNTs in PVA hydrogel matrix. **c**, Representative SEM images at the cross-section of untrained PVA hydrogel film and TRAIN CNTs (0.24 wt%)-PVA hydrogel film. Scale bars: 2 μm. **d**, Specific impedance of CNTs-PVA hydrogel fibers with different CNTs concentrations (n=5 individual CNTs-PVA hydrogel fibers). One-way ANOVA was used to determine statistical significance: $F_{3,16}=34.75$, ****$p<0.0001$. TEM images display CNTs at different concentrations. Scale bars: 50 nm. **e**, Comparative normalized impedance analysis of CNTs-PVA hydrogels with different CNTs concentrations across varied conditions (One-way ANOVA, 0.04 wt% CNTs: $F_{2,11}=0.0799$, non-significant (n.s.). 0.08 wt% CNTs: $F_{2,11}=12.07$, **$p=0.0017$. 0.16 wt% CNTs: $F_{2,11}=11.91$, **$p=0.0018$. 0.24 wt% CNTs: $F_{2,11}=49.35$, ****$p<0.0001$. n=4 individual CNTs-PVA hydrogel fibers and n=5 individual TRAIN hydrogel fibers). **f**, Normalized impedance profiles of CNTs-PVA (0.16 wt%) hydrogel fibers subjected to various cyclic training conditions (5%, 10%, and 20% strain for 5,000 cycles. One-way ANOVA, 5% training: $F_{2,11}=10.94$, **$p=0.0024$; 10% training: $F_{2,11}=11.91$, **$p=0.0018$; 20% training: $F_{2,11}=6.342$, *$p=0.0147$. n=4 individual CNTs-PVA hydrogel fibers and n=5 individual TRAIN hydrogel fibers). **g**, Impedance ratio (pre-training/post-10% training) of CNTs-PVA hydrogels with different CNTs concentrations (One-way ANOVA, $F_{3,16}=5.798$, **$p=0.0070$, n=5 individual TRAIN hydrogel fibers). Inset: schematic of CNTs-PVA hydrogel fibers undergoing cyclic training in water. All the data are presented as mean ± standard deviation (s.d.). **h**, Normalized specific impedance of CNTs-PVA hydro microelectrodes under 20% strain training with different cycles.

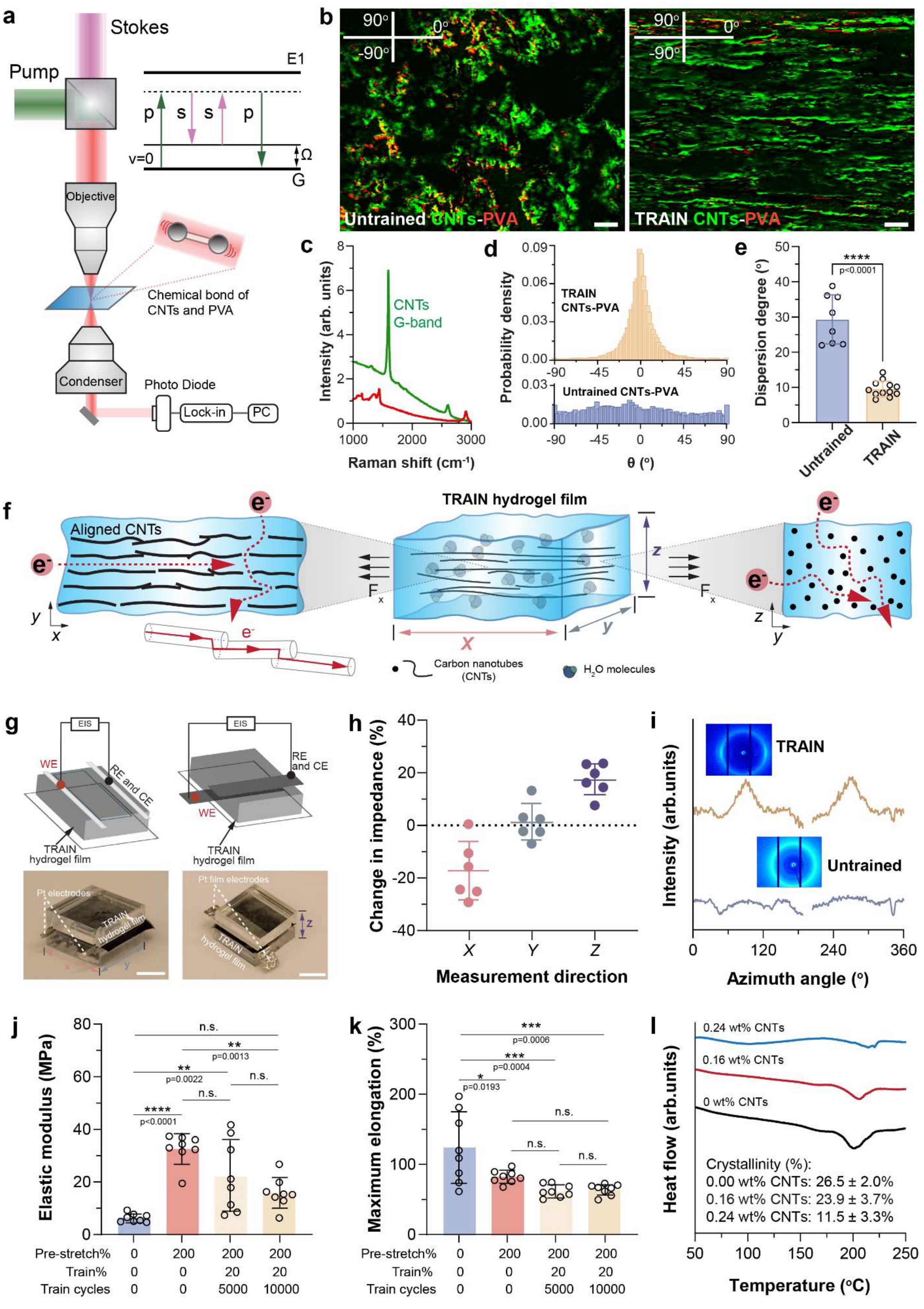

a
Stokes
Pump
E1
p s s p
v=0
Ω
G
Objective
Chemical bond of CNTs and PVA
Condenser
Photo Diode
Lock-in
PC
b
90°
0°
-90°
Untrained CNTs-PVA
TRAIN CNTs-PVA
c
Intensity (arb. units)
CNTs G-band
Raman shift (cm-1)
d
Probability density
TRAIN CNTs-PVA
Untrained CNTs-PVA
θ (°)
e
Dispersion degree (°)
****
p<0.0001
Untrained
TRAIN
f
Aligned CNTs
TRAIN hydrogel film
Carbon nanotubes (CNTs)
H2O molecules
g
EIS
WE
RE and CE
TRAIN hydrogel film
Pt electrodes
Pt film electrodes
h
Change in impedance (%)
Measurement direction
i
Intensity (arb.units)
TRAIN
Untrained
Azimuth angle (°)
j
Elastic modulus (MPa)
n.s.
**
p=0.0013
p=0.0022
****
p<0.0001
Pre-stretch% 0 200 200 200
Train% 0 0 20 20
Train cycles 0 0 5000 10000
k
Maximum elongation (%)
***
p=0.0006
***
p=0.0004
*
p=0.0193
n.s.
Pre-stretch% 0 200 200 200
Train% 0 0 20 20
Train cycles 0 0 5000 10000
l
0.24 wt% CNTs
0.16 wt% CNTs
0 wt% CNTs
Heat flow (arb.units)
Crystallinity (%):
0.00 wt% CNTs: 26.5 ± 2.0%
0.16 wt% CNTs: 23.9 ± 3.7%
0.24 wt% CNTs: 11.5 ± 3.3%
Temperature (°C)

685

**Figure 2**. **Mechanisms of TRAIN strategy. a**, Illustration of the Stimulated Raman Scattering (SRS) microscopy setup, highlighting the path of the laser, the sample interaction, and signal detection components. **b**, Comparative two-color SRS images of untrained CNTs (0.16 wt%)-PVA hydrogel and TRAIN CNTs (0.24 wt%)-PVA hydrogel. Scale bars: 20 μm. **c**, Raman spectra displaying the characteristic G-band of CNTs within the TRAIN CNT (0.16 wt%)-PVA hydrogel film compared to the PVA hydrogel film. **d**, Representative probability of CNTs orientations calculated from **b**. **e**, Dispersion degrees of untrained CNTs (0.16 wt%)-PVA hydrogel and TRAIN CNTs (0.24 wt%)-PVA hydrogel. Data are presented as mean ± s.d., n=8 individual hydrogel samples for untrained group and n=12 individual hydrogel samples for TRAIN group. Two-tailed unpaired student's t-test was used to determined statistical analysis: $F_{7,11}=9.794$, $t=9.216$, $df=18$, ****$p<0.0001$. **f**, Conductivity mechanisms in TRAIN CNTs-PVA hydrogel film along the *x, y,* and *z* directions, respectively. **g**, Left: A schematic of electrochemical characterization of TRAIN CNTs-PVA hydrogel film in *x* and *y* directions and an image of the characterization device. Scale: 1mm. Right: An illustration of electrochemical characterization of TRAIN CNTs-PVA hydrogel film in *z* direction and an image of characterization device. Scale: 1mm. WE: working electrode, RE: reference electrode, CE: counter electrode. **h**, Specific impedance changes (post-training/pre-training) of TRAIN CNTs-PVA hydrogels in *x*, *y*, *z* direction. **i**, Wide angle x-ray spectroscopy (WAXS) patterns of pre and post 20% trained CNTs-PVA hydrogel fibers. The break at 180° indicates the beam splits from the instrument. **j**, Elastic modulus of untrained CNTs (0.16 wt%)-PVA hydrogel fibers and TRAIN CNTs (0.16 wt%)-PVA hydrogel fibers. Data are presented as mean ± s.d., n=8 individual hydrogel fibers. One-way ANOVA was used to determined statistical analysis: $F_{3, 28}=15.36$, ****$p<0.0001$. **k**, Maximum elongation of untrained CNTs (0.16 wt%)-PVA hydrogel fibers and TRAIN CNTs (0.16 wt%)-

PVA hydrogel fibers. Data are presented as mean ± s.d., n=8 individual hydrogel fibers. One-way ANOVA was used to determined statistical analysis: $F_{3, 28}$=9.364, ***p=0.0002. **l**, Differential scanning calorimetry (DSC) curve of PVA and CNTs-PVA (0.16 wt% and 0.24 wt%) materials.

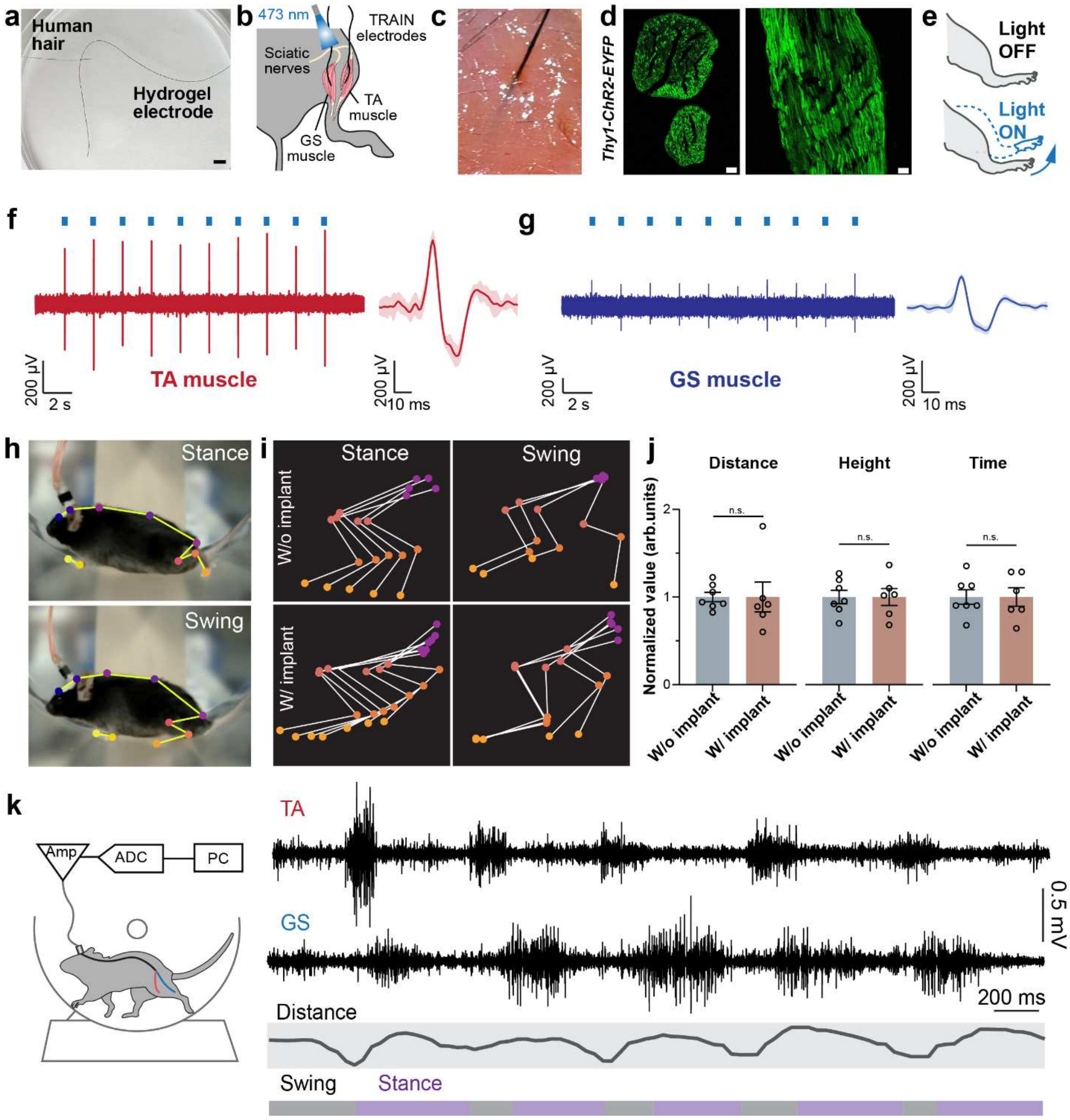

**Figure 3**. **TRAIN-enabled freely moving Electromyographic (EMG) recordings. a,** Image of a TRAIN microelectrode in water, being manipulated by a single human hair to demonstrate its delicate structure and flexibility. Scale: 5 mm. **b**, Schematic of TRAIN microelectrode implantation within the tibialis anterior (TA) and gastrocnemius (GS) muscles of a mouse. **C**, Representative visualization of a TRAIN microelectrode inserted into the GS muscle. **d**, Confocal image of sciatic nerve cross-sections (coronal and longitudinal) showing *Thy1-ChR2-EYFP* expression. Scale bars: 50 µm. **e**, A schematic of hindlimb kinematics of a *Thy1-ChR2-EYFP* mouse with and without optical stimulation. **f-g**, EMG recordings from the TA and GS muscles from a *Thy1-ChR2-EYFP* mouse with overlaid electrical signals corresponding to induced muscle contractions. Shadows present as mean ± s.d., blue bars indicate illumination cycles from a 473 nm laser. **h**, Pose estimation showing stance and swing phases from a mouse implanted with TRAIN microelectrodes in the TA and GS muscle during locomotion on a running wheel. **i**, Comparative gait analysis using pose estimation between a control mouse (without implant) and an experimental mouse (with implant). **j**, Quantitative evaluation of running distance, hindlimb retraction height, and duration for both non-implanted and implanted mice. Data are presented as mean ± standard error of the mean (s.e.m.), n=7 individual running cycles for non-implanted mouse and n=6 individual running cycles for implanted mouse. **k**, A schematic showing a mouse on a running wheel, with a TRAIN hydrogel electrode implanted in the TA muscle to capture EMG signals during free locomotion. Example of EMG recordings of the left TA and GS muscles from a mouse on a running wheel.

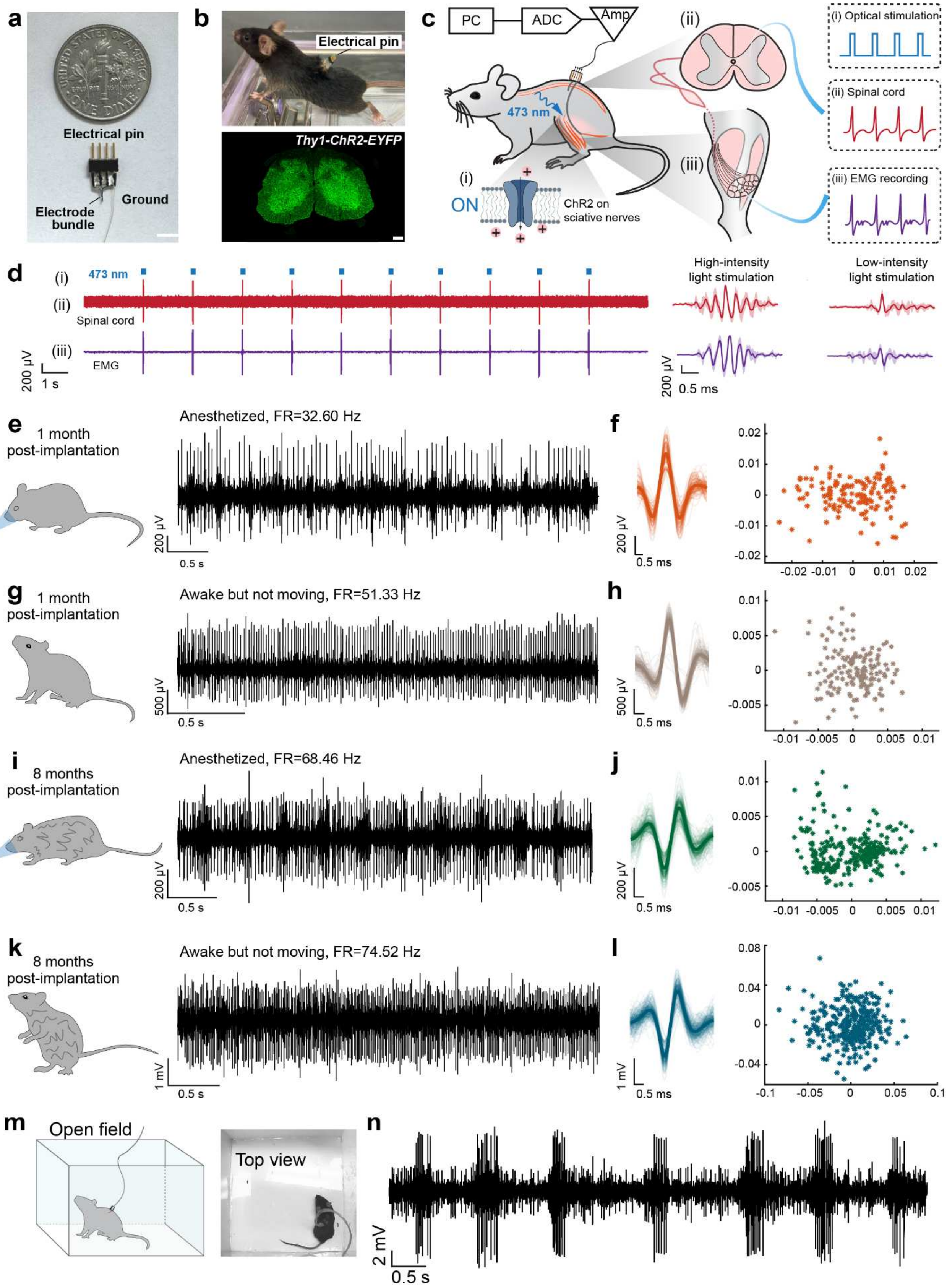

a
Electrical pin
Ground
Electrode bundle
b
Electrical pin
Thy1-ChR2-EYFP
c
PC
ADC
Amp
473 nm
(i)
ON
ChR2 on sciative nerves
(ii)
(iii)
(i) Optical stimulation
(ii) Spinal cord
(iii) EMG recording
d
(i) 473 nm
(ii) Spinal cord
(iii) EMG
200 μV
1 s
High-intensity light stimulation
Low-intensity light stimulation
200 μV
0.5 ms
e
1 month post-implantation
Anesthetized, FR=32.60 Hz
200 μV
0.5 s
f
200 μV
0.5 ms
g
1 month post-implantation
Awake but not moving, FR=51.33 Hz
500 μV
0.5 s
h
500 μV
0.5 ms
i
8 months post-implantation
Anesthetized, FR=68.46 Hz
200 μV
0.5 s
j
200 μV
0.5 ms
k
8 months post-implantation
Awake but not moving, FR=74.52 Hz
1 mV
0.5 s
l
1 mV
0.5 ms
m
Open field
Top view
n
2 mV
0.5 s

733

**Figure 4**. **Spinal cord electrophysiology with TRAIN microelectrodes. A,** Image of an assembled microelectrode probe for implantation into the spinal cord. Scale: 5 mm. **b**, Top: *Thy1-ChR2-EYFP* mouse post-operative with an implanted probe targeting the Lumbar (L) 3 region of the spinal cord. Bottom: Confocal microscopy image of a transverse section of the spinal cord L3 region exhibiting *Thy1-ChR2-EYFP* expression. **c**, Schematic illustration of the integrated neuromodulation system in a *Thy1-ChR2-EYFP* mouse, depicting an electronic device implanted at the L3 segment of the spinal cord with concurrent hydrogel electrode implantation in the hindlimb muscle. This multimodal system comprises synchronized components: (i) optical neural stimulation, (ii) electrophysiological recording from the spinal cord, and (iii) EMG from the hindlimb muscle. **d**, Representative recordings of spinal cord electrophysiological and EMG activity elicited by transdermal optical stimulation in *Thy1-ChR2-EYFP* mice. Overlaid traces (n=10 individual events, mean ± s.d.) under high- and low-intensity light stimulation illustrate the response dynamics. **e**, Endogenous spinal cord neural activity recorded from a mouse 1-month post-implantation under anesthesia. **f**, Overlaid wave forms of single neuron spiking from e and principal component analysis (PCA) from e showing a cluster of the overlaid waveforms. **g**, Spontaneous spinal cord neural activity recorded from an awake mouse 1-month post-implantation. h, Overlaid wave forms of single neuron spiking from g and PCA from g showing a cluster of the overlaid waveforms. **i**, Endogenous spinal cord neural activity recorded from a mouse 8 months post-implantation under anesthesia. **j**, Overlaid wave forms of single neuron spiking from i and PCA from i showing a cluster of the overlaid waveforms. **k**, Spontaneous spinal cord neural activity recorded from a conscious mouse 8 months after implantation **l**, Overlaid single neuron spike waveforms from **k**, with PCA from **k** illustrating a cohesive cluster. **m**, A schematic and an image of a mouse with an electronic device implanted in the spinal cord L3. **n**, Spontaneous neural activity traces of a moving mouse from spinal cord L3 in an open field.

## Supplementary Files

This is a list of supplementary files associated with this preprint. Click to download.

- AnisotropicHydrogelMicroelectrodesSI.docx
